# Supplementary material for: Learning the non-equilibrium dynamics of Brownian movies
Source: Nat Commun. 2020 Oct 23;11:5378. doi: 10.1038/s41467-020-18796-9 (PMC7585442; doi:10.1038/s41467-020-18796-9)
Supplement: Supplementary file 1 — Supplementary Information [file 41467_2020_18796_MOESM1_ESM.pdf]

Supplementary Information
Learning the Non-Equilibrium Dynamics of Brownian Movies

Federico S. Gnesotto and Grzegorz Gradziuk
Arnold-Sommerfeld-Center for Theoretical Physics and Center for NanoScience, Ludwig-Maximilians-Universität München, D-80333 München, Germany.

Pierre Ronceray*
Center for the Physics of Biological Function, Princeton University, Princeton, NJ 08544, USA

Chase P. Broedersz*
Arnold-Sommerfeld-Center for Theoretical Physics and Center for NanoScience, Ludwig-Maximilians-Universität München, D-80333 München, Germany. and Department of Physics and Astronomy, Vrije Universiteit Amsterdam, 1081 HV Amsterdam, The Netherlands

* ronceray@princeton.edu, c.broedersz@lmu.de

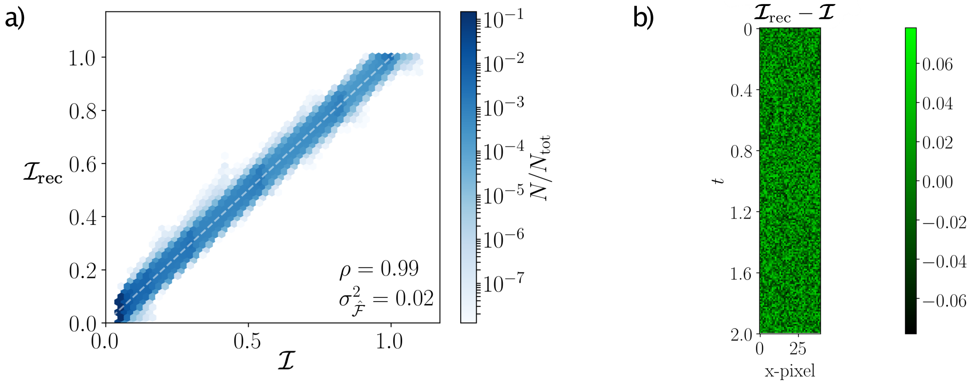

Supplementary Figure 1. **Two-beads Brownian movie: Comparison between reconstructed and exact images** Here we compare the exact two-beads images $\boldsymbol{\mathcal{I}}$ to the reconstructed images $\boldsymbol{\mathcal{I}}_{\rm rec}$ using PCA (see main text Fig. 2d). We do so with a scatter plot of the pixel values at different time points and with a kymograph of the difference between exact and reconstructed images, as shown in Supplementary Figure 1. Overall, we find that the first four PCA modes allow for an accurate reconstruction of the images in the Brownian movie for this two-beads model. a) Scatter plot of reconstructed (with 4 principle components) image-pixel values $\boldsymbol{\mathcal{I}}_{\rm rec}$ and exact image-pixel values $\boldsymbol{\mathcal{I}}$ using PCA. The Pearson correlation coefficient ρ and the relative squared error $\sigma^2_{\boldsymbol{\mathcal{I}}}$ (see main text for definition) are shown. Data is the same as in Fig. 2d of the main text. b) Kymograph of the difference between reconstructed (with 4 principle components) and exact pixel values along the horizontal line shown in Fig. 2d of the main text.

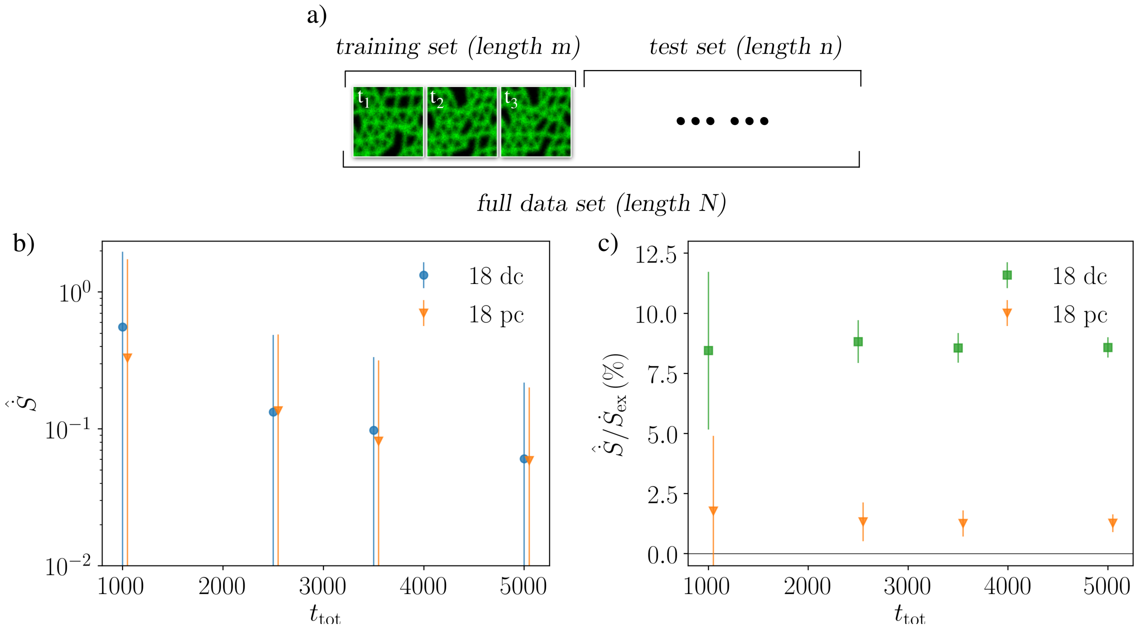

Supplementary Figure 2. **Dependence of Entropy Production Rates on the trajectory length** a): Schematic of the training/test set splitting procedure: the full trajectory (length N) is split into a training set (length m) and into a test set (length n). b) Decay to zero of the entropy production rate bias (estimated with 18 principled components (pc)-blue dots and 18 principled components (pc)-orange triangles) as a function of the trajectory length at equilibrium. c) Convergence of the entropy production rate (estimated with 18 pc-orange triangles and 18 dc-green squares) as a function of the trajectory length. The error bars in panels b,c) represent an estimate of the root-mean-square deviation between the true apparent entropy production rate and the inferred value (see Methods). The parameters of the simulations and noise level are the same as in Fig. 3 of the main text. Equilibrium is obtained by setting all temperatures equal to $T_0 = 0.05$.

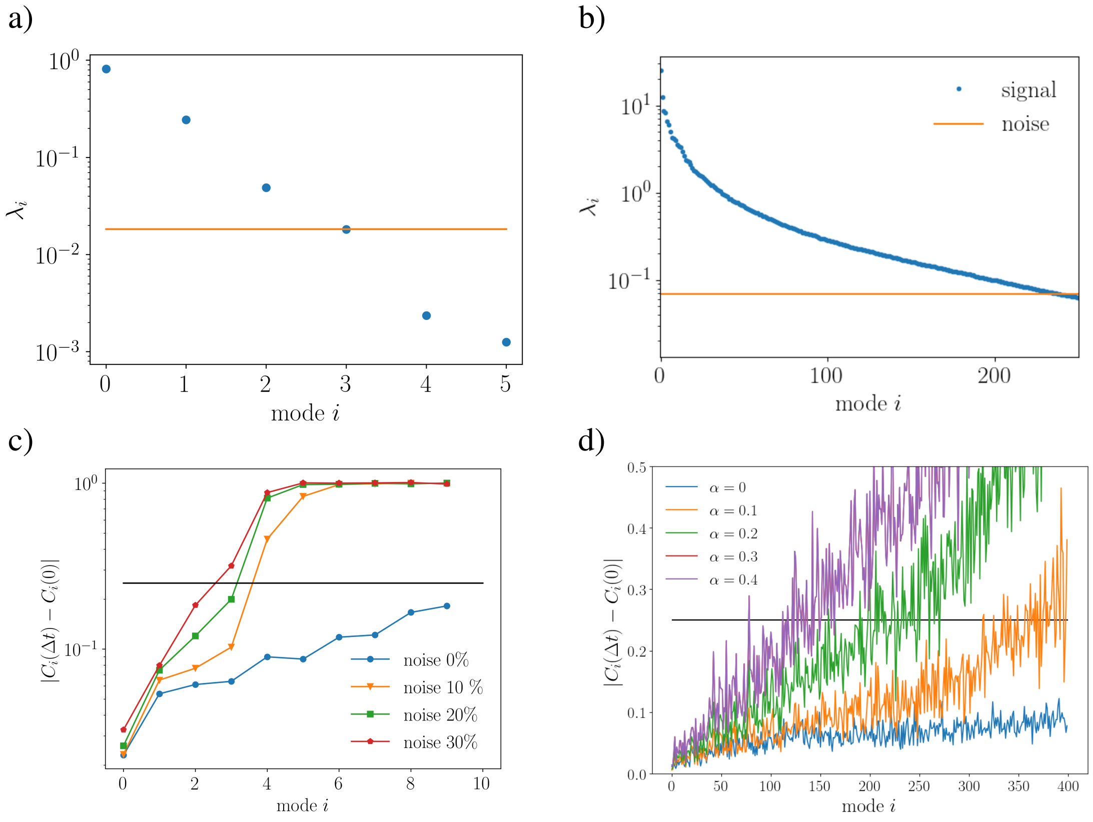

Supplementary Figure 3. **Detecting the noise floor** a-b): Eigenvalues λ_i of the covariance matrix for the data $\boldsymbol{X}$ (blue markers) together with the noise floor (largest eigenvalue of $\boldsymbol{X}_{\text{shuffled}}$-orange line) for the two-beads model (a) and the focus-region of the 20×20 network analyzed in Fig. 3 of the main text (b). In panel (a) the noise level on the image is 10%, in panel (b) $\alpha = 0.1$. c-d): Decrease (absolute value) of the autocorrelation function of principal component coefficients after one time-step at different noise levels for the two-bead model (c) and the focus-region of the filamentous network (d). The solid line indicates the 25% level used in our criterion. Panels a,c (Panels b,d): same simulation parameters as Fig. 2 (Fig. 3) of the main text.

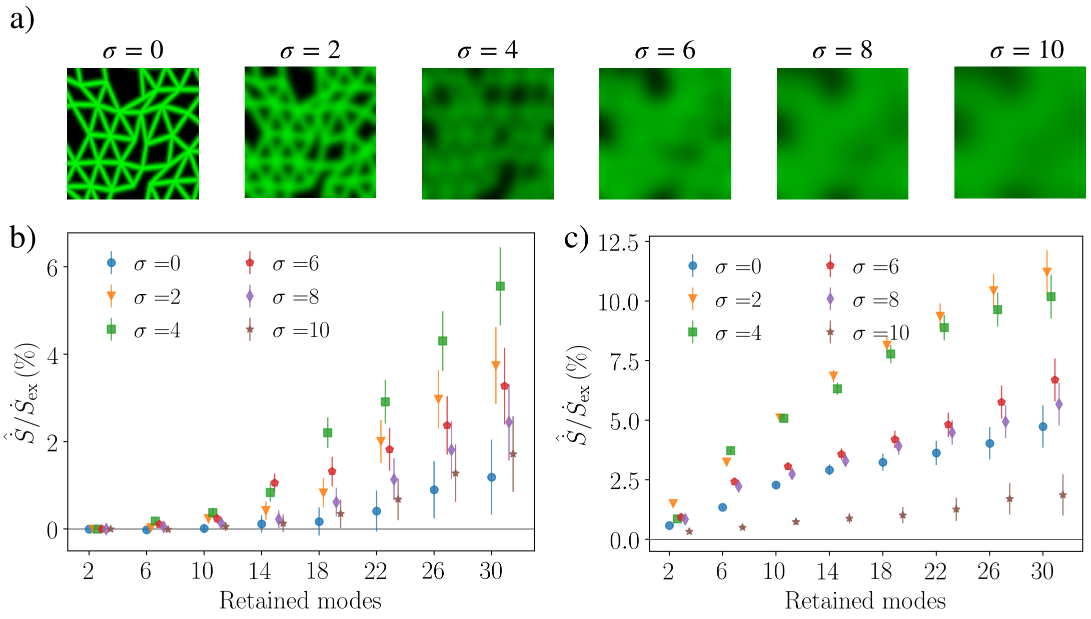

Supplementary Figure 4. **Inferring entropy production rates of Brownian movies with image blurring** a) Image-frames (80×80) of the analyzed patch for different values of the blurring parameter σ. b-c) Percentage of recovered entropy production rate $\widehat{\dot{S}}/\dot{S}_{\rm ex}$ vs retained modes with PCA (b) and DCA (c) for varying σ. The error bars represent an estimate of the root-mean-square deviation between the true apparent entropy production rate and the inferred value (see Methods). All results have been obtained for a trajectory of 10^6 time steps, $\Delta t = 0.005$ and 80×80 frames. DCA was performed on the first 200 principle components for $\sigma = 2$, on the first 100 principle components for $\sigma = 4$, on the first 50 principle components for $\sigma = 6$, on the first 40 principle components for $\sigma = 8$, and on the first 34 principle components for $\sigma = 10$. Stochastic Force Inference was used with a first order polynomial basis for the inference of the diffusion tensor and of the force field. The noise-corrected diffusion estimator was employed.

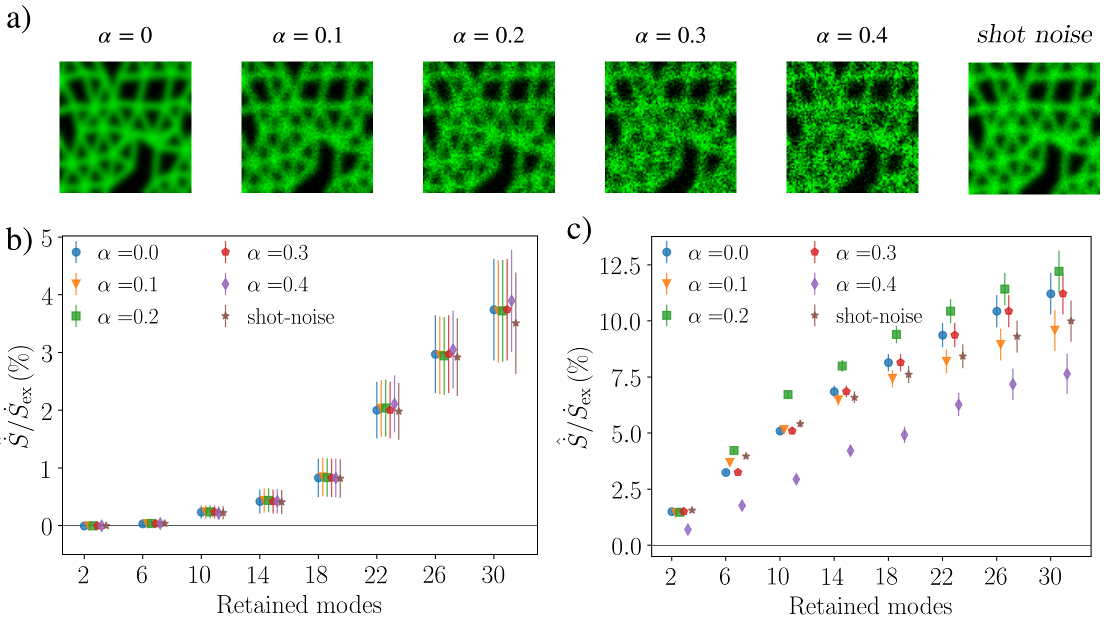

Supplementary Figure 5. **Inferring entropy production rates of Brownian movies with imaging noise** a) Image-frames (80×80) of the analyzed patch for different values of the white gaussian noise parameter α and for shot noise. The built-in python-numpy functions for generation of normal and poisson distributed numbers are employed to generate the noise. b-c) Percentage of recovered entropy production rate $\widehat{\dot{S}}/\dot{S}_{\mathrm{ex}}$ vs retained modes with PCA (b) and DCA (c) for varying α and for shot noise. The error bars represent an estimate of the root-mean-square deviation between the true apparent entropy production rate and the inferred value (see Methods). All results have been obtained for a trajectory of 10^6 time steps, $\Delta t = 0.005$ and 80×80 frames. DCA was performed on the first 200 principle components for $\alpha = 0, 0.1$ and for the shot noise case, on the first 150 principle components for $\alpha = 0.2$, on the first 100 principle components for $\alpha = 0.3$, on the first 50 principle components for $\alpha = 0.4$. Stochastic Force Inference was used with a first order polynomial basis for the inference of the diffusion tensor and the force field. The noise-corrected diffusion estimator was employed.

SUPPLEMENTARY NOTE 1
NUMERICALLY INTEGRATING THE BROWNIAN DYNAMICS

We simulate the stochastic dynamics of the two-beads model by numerically integrating the overdamped Langevin equation for the beads' displacements $\mathbf{x} = (x_1, x_2)$ (Eq. 1 main text), with $\mathbf{F}(\mathbf{x}) = \boldsymbol{K}\mathbf{x}$, $K_{ij} = (1-3\delta_{ij})k/\gamma$, $D_{ij} = \delta_{ij}k_{\mathrm{B}}T_i/\gamma$. We discretize the equation of motion for the two beads using an Euler scheme with discretization step Δt. Thus, the discretized equation of motion after n time steps for the i-th bead reads: $x_i((n+1)\Delta t) = x_i(n\Delta t) + \sum_{j=1,2} K_{ij}x_j(n\Delta t)\Delta t + \sum_{j=1,2}\sqrt{2D_{ij}\Delta t}\xi_j$, where ξ_j is a random number drawn from a normal distribution with mean zero and variance one. We initialize the simulation with the beads in their rest state and we only record the positions of the beads after an equilibration time $t_{\mathrm{eq}} = 10^5\Delta t$ to allow the dynamics to reach steady state. The parameters for the results of Fig. 2 of the main text are: $\Delta t = 0.01$, $k = 2$, $\gamma = 1$, $k_B = 1$, $T_1 = 1$ and $0.2 < T_2 \leq 1$.

The dynamics of the 20×20 spring network is generated in a similar way. In this case, we discretize the overdamped Langevin equation for the nodes' positions $\mathbf{x}$ with time step $\Delta t = 0.005$. For the network the elastic force acting on node i reads: $\mathbf{F}_i(\mathbf{x}) = -\sum_{j\sim i}\frac{k_{ij}}{\gamma}(\|\mathbf{x}_{i,j}(t)\| - \ell_0)\hat{\mathbf{x}}_{i,j}$, where $k_{ij} = k$ if the bond is present, $k_{ij} = 0$ if it is not, $\mathbf{x}_{i,j} = \mathbf{x}_i - \mathbf{x}_j$ is the unit vector between nodes i and j, the sum runs over nearest-neighbor nodes j of i, $k = 4$, and $\ell_0 = \gamma = k_B = 1$. In Fig. 3 of the main text, we randomly choose $1/5$ of the nodes to have higher temperature $T_{\mathrm{hot}} = 0.25$, while the rest has a lower temperature $T_{\mathrm{cold}} = 0.05$ ($T_{\mathrm{hot}} = 0.05$ and $T_{\mathrm{cold}} = 0.01$ in the low temperature case). Additionally, we randomly dilute the bonds of the network with probability $1/5$. The simulation is initialized with the network in its rest state and we wait an equilibration time $t_{\mathrm{eq}} = 10^5\Delta t$ before recording trajectories.

SUPPLEMENTARY NOTE 2
GENERATING THE BROWNIAN MOVIES

We first outline the procedure to generate a Brownian movie for the two-beads model (see Fig. 2 of the main text). The input consists of the numerically generated position trajectories of the two beads. We then transform the trajectories from position space to image space into pixel units (we used a 40×20 pixel grid). Specifically, we set the image pixel intensities at a given time point by centering a radially symmetric Gaussian function centered at the bead's position, with amplitude 1 and variance 9 pixels. Finally, to simulate measurement noise in a simple way, we add uncorrelated white noise sampled uniformly from $[0, a]$ ($a = 0.1$, i.e. in Fig. 2 of the main text) independently at each pixel. As in real imaging devices, pixels are saturated at intensity 1.

Next, we briefly detail how we generate a movie for a region inside a 20×20 network. The $N \times 800$-dimensional position array (N is the number of recorded time steps), which is the output of the numerical integration of the Langevin equation, is transferred to a custom Python routine that, at each time step, directly plots all the lines connecting neighboring nodes within the selected smaller region (grey frame of Fig. 3a of the main text) onto a 80×80 pixel grid. In this routine, pixel intensities decay with the distance from each such line as a Gaussian function with amplitude 0.8 and a variance of 2 pixels. Additionally, to simulate limited optical resolution of the imaging device, we blur the images as described in Supplementary Note 6. To simulate measurement noise, we add white gaussian noise as described in Supplementary Note 7. Note, from our Brownian dynamics simulation we can compute the exact entropy production rate (in natural units with $k_{\mathrm{B}} = 1$) $\dot{S}_{\mathrm{tot}} = 786$ of the full 20×20 network. To estimate the exact entropy production rate $\dot{S}_{\mathrm{ex}}$ of the observed region in the Brownian movie, we assume the spatial density of entropy production rate to be approximately uniform throughout the whole 20×20 network. The exact entropy production rate for the observed region is then $\dot{S}_{\mathrm{ex}} \approx \dot{S}_{\mathrm{tot}}A_{\mathrm{obs}}/A_{\mathrm{tot}}$, where $A_{\mathrm{tot}} = 381.5$ is the area of the full network measured in natural units, and $A_{\mathrm{obs}} = 24.5$ the area of the observed region. From this we obtain $\dot{S}_{\mathrm{ex}} = 786 \times 24.5/381.5 \approx 50.5$ for the network, which is the value we employ throughout the manuscript.

SUPPLEMENTARY NOTE 3
INFERRING THE DISSIPATIVE MODES: DISSIPATIVE COMPONENT ANALYSIS

The aim of Dissipative Component Analysis (DCA) is to infer a set of modes that maximize dissipation or, more precisely, the entropy production rate. This method is a principled approach only for a linear dynamical

system with constant diffusion. However, as we demonstrate in the main text, this method can be successfully employed in high-dimensional situations when dealing with image-data, when the dynamics is close to linear (close to the stable fixed points of the system). In such cases DCA can reduce the dimensionality by exploiting the non-equilibrium character of the system, as outlined below.

We consider a generic linear system described by an n-dimensional column-vector of coordinates $\mathbf{y}$ that obeys the Langevin equation

$$\frac{d\mathbf{y}(t)}{dt} = \boldsymbol{K}\mathbf{y}(t) + \sqrt{2\boldsymbol{D}}\boldsymbol{\xi}(t)\,, \tag{1}$$

where $\boldsymbol{K}$ is the interaction matrix and $\boldsymbol{D}$ the diffusion matrix. Note that $\boldsymbol{D}$ and $\boldsymbol{K}$ may in general not satisfy detailed balance and the system may thus be out of equilibrium.

As a preliminary step we perform principal component analysis (PCA) on data obtained simulating the time-evolution described by Eq. 1 for N time-steps: we first compute the covariance matrix $\boldsymbol{C} = \frac{1}{N}\sum_{t=1}^{N}(\mathbf{y}(t) - \langle\mathbf{y}\rangle)\cdot(\mathbf{y}^T(t) - \langle\mathbf{y}\rangle^T)$, where $\langle\mathbf{y}\rangle = \frac{1}{N}\sum_{t=1}^{N}\mathbf{y}(t)$. We then retain the first $m < n$ eigenvectors of $\boldsymbol{C}$ (see Supplementary Note 5 for details on the truncation criteria), ordered by magnitude of the associated eigenvalues, and use them to construct the $m \times n$ transformation matrix $\boldsymbol{E}$. The time evolution of the system projected onto the PC-coordinates is then $\mathbf{y}_{\text{pca}}(t) = \boldsymbol{E}^T\mathbf{y}(t)$. In this basis, the covariance matrix $\boldsymbol{C}_{\text{pca}}$ is diagonal with the ordered eigenvalues as diagonal entries. This preliminary step is useful for two reasons: it reduces dimensionality and it conveniently filters out measurement noise from the images. Next, we transform the data into covariance identity coordinates (cic), in which the covariance matrix is the identity. This is accomplished by $\mathbf{y}_{\text{cic}}(t) = \boldsymbol{C}_{\text{pca}}^{-1/2}\boldsymbol{E}^T\mathbf{y}(t)$.

In the next step, we focus on the non-equilibrium character of the system and compute the area-enclosing-rate matrix (AER) $\dot{\boldsymbol{A}}$ in CIC coordinates [1–3]:

$$\dot{A}_{\text{cic},ij} = \frac{1}{2t_{\text{tot}}}\sum_{t=1}^{N}[y_{\text{cic},i}(t)\Delta y_{\text{cic},j}(t) - y_{\text{cic},j}(t)\Delta y_{\text{cic},i}(t)], \tag{2}$$

where $t_{\text{tot}} = N\Delta t$ is the total simulation time and Δy_i denotes the displacement of the i-th coordinate between two successive time-steps. Each element $\dot{\boldsymbol{A}}_{ij}$ of the AER matrix corresponds to the area that the trajectory encloses on average in the plane (y_i, y_j) per unit time. This area enclosing rate quantifies broken detailed balance in the system and is zero in thermal equilibrium. Having defined the AER allows us to conveniently write the total entropy production of the system as [2, 4]:

$$\dot{S} = \text{Tr}(\dot{\boldsymbol{A}}_{\text{cic}}\dot{\boldsymbol{A}}_{\text{cic}}^T\boldsymbol{D}_{\text{cic}}^{-1}), \tag{3}$$

where $\boldsymbol{D}_{\text{cic}} := \frac{1}{2t_{\text{tot}}}\sum_t \Delta\mathbf{y}_{\text{cic}}(t)\Delta\mathbf{y}_{\text{cic}}^T(t)$. It is now key to observe that the matrix product $\dot{\boldsymbol{A}}_{\text{cic}}\dot{\boldsymbol{A}}_{\text{cic}}^T$, appearing in the expression for the entropy production rate Eq. 3, is real and symmetric and thus admits a real orthonormal basis of eigenvectors. Moreover, since $\dot{\boldsymbol{A}}_{\text{cic}}$ is antisymmetric, all non-zero eigenvalues of $\dot{\boldsymbol{A}}_{\text{cic}}\dot{\boldsymbol{A}}_{\text{cic}}^T$ are two-fold degenerate. Furthermore, note that the orthonormal basis of $\dot{\boldsymbol{A}}_{\text{cic}}\dot{\boldsymbol{A}}_{\text{cic}}^T$ is unique up to rotations in the two-dimensional eigenspaces that correspond to the same eigenvalue. Importantly, in these special covariance identity coordinates (scic), the total entropy production rate reads

$$\dot{S} = \sum_{i\in odd}\lambda_i[(D_{\text{scic}}^{-1})_{ii} + (D_{\text{scic}}^{-1})_{i+1\,i+1}], \tag{4}$$

with λ_i being the eigenvalues of $\dot{\boldsymbol{A}}_{\text{cic}}\dot{\boldsymbol{A}}_{\text{cic}}^T$. We refer to the corresponding eigenvectors as the dissipative components.

SUPPLEMENTARY NOTE 4
DEPENDENCE OF ENTROPY PRODUCTION RATES ON THE TRAJECTORY LENGTH

The entropy production rate is a semi-positive definite quantity: at steady state $\dot{S} \geq 0$. Given finite-length data, the estimate of the entropy production rate will be biased. While this bias can be computed analytically for homogeneous diffusion coefficients [5], this may be difficult for space-dependent diffusion coefficients and in the presence of measurement noise. Given that we are here concerned with finite-size data of systems

with multiplicative noise partially corrupted by measurement noise, we use the following approach to reduce the bias of the entropy production rate and, correspondingly, to avoid overfitting: We separate our data set of length N into two independent and successive sets, a training set of length m and a test set of length $n = N - m$. The results in Fig. 3 of the main text are obtained with $m = N/10$. We first infer relevant components using the training set, and we then project the test set onto these components and infer the corresponding entropy production rate, as shown in Supplementary Figure 2a . Although entropy production rate estimates remain weakly positively biased for short trajectories, the bias approaches zero for long trajectories, as shown in Supplementary Figure 2b for the analyzed region of the 20×20 spring network with uniform temperatures (equilibrium). Note, however that our error bar estimates always intersect zero for all trajectory lengths (Supplementary Figure 2b). When the network is out of equilibrium, the entropy production rate estimates converge to non-zero values for long trajectories, as shown in

SUPPLEMENTARY NOTE 5
DIMENSIONAL REDUCTION: TRUNCATION CRITERIA

For the Brownian-movie learning procedure it is important to reduce the dimensionality of image data to a more tractable number of components. Therefore, we require criteria to decide on the maximum number of components that we consider in our analysis of the stochastic dynamics. Two main limiting effects arise due to the finite length of trajectories and measurement noise.

1) Noise floor

We start by asking what is the maximum number of components that we can distinguish from a noise floor set by the imaging noise and the finite length of the data. Our image data is a matrix $\boldsymbol{X}$ of t_{tot} (total simulation time) rows and $L \times W$ (total number of pixels in a single image) columns. We first estimate the principal components — the normalized eigenvectors of the covariance matrix of image data — and sort these components according to the magnitude of the corresponding eigenvalues. To determine the noise floor, we eliminate temporal correlations in the image data by shuffling the values of $\boldsymbol{X}$ separately along each of its columns [6]. What we obtain is a shuffled data set $\boldsymbol{X}_{\text{shuffled}}$ for which we can also compute principal components and eigenvalues. The largest eigenvalue of the covariance matrix of $\boldsymbol{X}_{\text{shuffled}}$ yields the noise floor. Thus, we truncate the basis of principal components to exclude components with eigenvalues below this noise floor. To illustrate this procedure, a plot of the eigenvalues for $\boldsymbol{X}$ together with the noise threshold is shown in Supplementary Figure 3 a-b for the two beads model and for the filamentous network.

2) Resolution of the dynamics

Criterion 1) ensures that the components are distinguishable from imaging noise, which is a static property of the data. The Brownian-movie analysis is concerned with the dynamics. We thus want to make sure that we can resolve the dynamics of the components selected with criterion 1). This is a necessary condition to infer force and diffusion fields in image-space. A criterion for selecting components whose dynamics can be resolved using SFI is based on computing the autocorrelation function of the projection coefficients ($\mathbf{c}$ in the main text) centered around their average value ($c_i(t) \to c_i(t) - \langle c_i \rangle$):

$$C_i(n\Delta t) = \frac{\sum_{t=1}^{N-n\Delta t} c_i(t+n\Delta t) c_i(t)}{\sum_{t=1}^{N} c_i^2(t)} \,. \tag{5}$$

We are only able to resolve the dynamics if $c_i(t)$ does not decorrelate too fast, i.e. if $C_i(n\Delta t)$ does not decay to zero in a time comparable to the time-step Δt. We therefore employ the following criterion: we only retain components for which $|C_i(\Delta t) - C_i(0)| < 0.25$. We applied criterion 2) to the two-beads data and to the network data and plot the results in Supplementary Figure 3 c-d. Criterion 2) is clearly sensitive both to the time resolution Δt and to the signal to noise ratio in the trajectories.

SUPPLEMENTARY NOTE 6
ENTROPY PRODUCTION RATE INFERENCE FOR INCREASING IMAGE-BLURRING

The imaging of a physical system will be subject to a finite resolution, which will induce image blurring. Here, we test the performance of our inference method as we increase the blurring of the object of interest in the Brownian movie, as shown in Supplementary Figure 4a. Blurring of the original image (σ=0) is obtained by applying the standard gaussian filter of the scikit-image python library. The blurring parameter σ corresponds to the standard deviation of the gaussian kernel. Our estimates of the recovered entropy production rates are affected by high levels of blurring of the structure of interest, as shown in Supplementary Figure 4b-c. Interestingly, when blurring is modest ($\sigma = 2, 4$) our method recovers more entropy production rate than without any blurring ($\sigma = 0$), possibly because the image-space dynamics is expected to become more linear with a slightly blurred system. If blurring is further increased and objects in the movie start to overlap ($\sigma > 4$), the $\widehat{\dot{S}}/\dot{S}_{\mathrm{ex}}$ estimates start to decline. Remarkably however, both PCA and DCA still yield non-zero estimates of $\widehat{\dot{S}}$ with very strong blurring $\sigma = 10$, if a sufficient number of modes ($\gtrsim 10$) are retained. Furthermore, for this example DCA outperforms PCA, also for blurred Brownian movies.

SUPPLEMENTARY NOTE 7
ENTROPY PRODUCTION RATE INFERENCE FOR INCREASING NOISE LEVEL

In this section, we test the robustness of our entropy production inference method to increasing levels of measurement noise in the image-frames. To simulate imaging noise, we consider two different white-noise contributions: we include an intensity-dependent contribution $\boldsymbol{\mathcal{N}}_{\boldsymbol{\mathcal{I}}}$, and a white gaussian noise $\boldsymbol{\mathcal{N}}_0$, which is independent of the intensity at each pixel. Thus, the intensity of each frame $\boldsymbol{\mathcal{I}}(t)$ is given by:

$$\boldsymbol{\mathcal{I}}(t) = \bar{\boldsymbol{\mathcal{I}}}(t) + \boldsymbol{\mathcal{N}}_{\boldsymbol{\mathcal{I}}}(t) + \boldsymbol{\mathcal{N}}_0(t), \tag{6}$$

where $\bar{\boldsymbol{\mathcal{I}}}$ is the ideal image, and the intensity-dependent white noise $\boldsymbol{\mathcal{N}}_{\boldsymbol{\mathcal{I}}}$ is drawn from a normal distribution of zero mean and standard deviation $\alpha\sqrt{\bar{\boldsymbol{\mathcal{I}}}(t) + \boldsymbol{\mathcal{N}}_0(t)}$ (negative values of $\bar{\boldsymbol{\mathcal{I}}}(t) + \boldsymbol{\mathcal{N}}_0(t)$ are truncated at zero), where $\boldsymbol{\mathcal{N}}_0(t)$ the background white noise term drawn independently at each time step from a normal distribution of zero mean and standard deviation $\alpha/10$. Negative pixel intensities are truncated at zero. Additionally, to simulate the case of shot noise in the imaging apparatus, we also consider additive Poisson-distributed noise with a mean and variance equal to the intensity at each pixel $\bar{\boldsymbol{\mathcal{I}}}(t)$.

Examples of noisy image frames are shown in Supplementary Figure 5a. The estimated entropy production rate via DCA and PCA are robust to modest levels of white gaussian noise in the image ($\alpha = 0.1, 0.2, 0.3$), as well as to shot noise, as shown in Supplementary Figure 5b-c. For $\alpha = 0.4$ we are probing the limit of our inference method (our truncation criteria allows us to perform DCA only with the first 50 principle components) and, although we are still able estimate a significant amount of entropy production, the performance decreases.

SUPPLEMENTARY NOTE 8
PROOF OF LOWER BOUND ON THE ENTROPY PRODUCTION RATE

A common problem in the inference of the entropy production rate is that we only observe a part of the system, which typically makes it impossible to infer the exact value of the total entropy production rate. However, even with hidden degrees of freedom one can still find a non-trivial lower bound to the total entropy production rate. Here we offer a proof to demonstrate this for a class of systems described by a Langevin equation with multiplicative Gaussian white noise.

Let us denote by $\boldsymbol{D}(\mathbf{x})$ the diffusion matrix. We split the system into observed part "o" and hidden part "h", such that the diffusion matrix has the form

$$\boldsymbol{D} = \begin{pmatrix} \boldsymbol{D}_{\mathrm{oo}} & \boldsymbol{D}_{\mathrm{oh}} \\ \boldsymbol{D}_{\mathrm{ho}} & \boldsymbol{D}_{\mathrm{hh}} \end{pmatrix} \tag{7}$$

Denoting by $\mathbf{v}(\mathbf{x}) = (\mathbf{v}_{\mathrm{o}}(\mathbf{x}), \mathbf{v}_{\mathrm{h}}(\mathbf{x}))$ the mean phase space velocity, by $\tilde{\mathbf{v}}_{\mathrm{o}}(\mathbf{x}_{\mathrm{o}}) = \langle \mathbf{v}_{\mathrm{o}}(\mathbf{x}) | \mathbf{x}_{\mathrm{o}} \rangle$ the apparent mean phase space velocity, and by $\tilde{\boldsymbol{D}}_{\mathrm{oo}}(\mathbf{x}_{\mathrm{o}}) = \langle \boldsymbol{D}_{\mathrm{oo}}(\mathbf{x}) | \mathbf{x}_{\mathrm{o}} \rangle$ the apparent diffusion matrix of the observed

subsystem, we define the apparent entropy production rate as

$$\dot{S}_{\text{app}} = \langle \tilde{\mathbf{v}}_{\text{o}} (\tilde{\boldsymbol{D}}_{\text{oo}})^{-1} \tilde{\mathbf{v}}_{\text{o}} \rangle \tag{8}$$

The total entropy production rate of the system is calculated as

$$\dot{S} = \langle \mathbf{v} \boldsymbol{D}^{-1} \mathbf{v} \rangle \tag{9}$$

We aim to show that $\dot{S}_{\text{app}} \leq \dot{S}$. The proof follows in two steps. First, given that the function $\pi(\boldsymbol{D}, \mathbf{v}) = \mathbf{v}\boldsymbol{D}^{-1}\mathbf{v}$ restricted to positive definite matrices $\boldsymbol{D}$ is multivariate convex (Lemma 1), the Jensen inequality implies:

$$\dot{S}_{\text{app}} = \langle \tilde{\mathbf{v}}_{\text{o}} (\tilde{\boldsymbol{D}}_{\text{oo}})^{-1} \tilde{\mathbf{v}}_{\text{o}} \rangle \leq \langle \mathbf{v}_{\text{o}} (\boldsymbol{D}_{\text{oo}})^{-1} \mathbf{v}_{\text{o}} \rangle \tag{10}$$

Second, we demonstrate that (Lemma 2):

$$\forall_{\mathbf{v} \in \mathbb{R}^n} \quad \mathbf{v}_{\text{o}} (\boldsymbol{D}_{\text{oo}})^{-1} \mathbf{v}_{\text{o}} \leq \mathbf{v} \boldsymbol{D}^{-1} \mathbf{v} \tag{11}$$

Put together, Lemma's 1 and 2 imply:

$$\dot{S}_{\text{app}} = \langle \tilde{\mathbf{v}}_{\text{o}} (\tilde{\boldsymbol{D}}_{\text{oo}})^{-1} \tilde{\mathbf{v}}_{\text{o}} \rangle \leq \langle \mathbf{v} \boldsymbol{D}^{-1} \mathbf{v} \rangle = \dot{S} \tag{12}$$

Proof of Lemma 1

Let $\mathbf{v} \in \mathbb{R}^n$ and $\boldsymbol{D} \in \mathbb{R}^{n^2}$. The function $\pi : \mathbb{R}^n \times \mathbb{R}^{n^2} \to \mathbb{R}$ is defined by $\pi(\mathbf{v}, \boldsymbol{D}) = \mathbf{v}\boldsymbol{D}^{-1}\mathbf{v} = v_\mu D^{-1}_{\mu\nu} v_\nu$, using the Einstein summation convention. Let $\boldsymbol{\mathcal{H}}^\pi[\mathbf{v}, \boldsymbol{D}]$ denote the Hessian of π at point $(\mathbf{v}, \boldsymbol{D})$. Let us also denote by $\mathcal{S}_n$ the space of symmetric matrices of size $n \times n$ and by $\mathcal{S}_n^+$ the subset of positive definite matrices. Note that $\mathcal{S}_n$ is a linear subspace of $\mathbb{R}^{n^2}$ and that $\mathcal{S}_n^+$ is a convex set.
We need to prove that $\pi|_{\mathbb{R}^n \times \mathcal{S}_n^+}$ is a convex function. Because $\mathcal{S}_n$ is a linear subspace of $\mathbb{R}^{n^2}$, it is enough to prove that at every point $(\mathbf{v}, \boldsymbol{D}) \in \mathbb{R}^n \times \mathcal{S}_n^+$ the Hessian $\boldsymbol{\mathcal{H}}^\pi[\mathbf{v}, \boldsymbol{D}]$ is positive semi-definite on $\mathbb{R}^n \times \mathcal{S}_n$, that is:

$$\forall_{(\mathbf{v}, \boldsymbol{D}) \in \mathbb{R}^n \times \mathcal{S}_n^+} \forall_{(\mathbf{w}, \boldsymbol{P}) \in \mathbb{R}^n \times \mathcal{S}_n} \quad (\mathbf{w}, \boldsymbol{P}) \boldsymbol{\mathcal{H}}^\pi[\mathbf{v}, \boldsymbol{D}](\mathbf{w}, \boldsymbol{P}) \geq 0 \tag{13}$$

In order to verify this condition we first calculate the full Hessian $\boldsymbol{\mathcal{H}}^\pi$ defined as:

$$\boldsymbol{\mathcal{H}}^\pi = \begin{pmatrix} \frac{\partial^2 \pi}{\partial \mathbf{v} \partial \mathbf{v}} & \frac{\partial^2 \pi}{\partial \mathbf{v} \partial \boldsymbol{D}} \\ \frac{\partial^2 \pi}{\partial \boldsymbol{D} \partial \mathbf{v}} & \frac{\partial^2 \pi}{\partial \boldsymbol{D} \partial \boldsymbol{D}} \end{pmatrix} := \begin{pmatrix} \boldsymbol{A} & \boldsymbol{B} \\ \boldsymbol{B}^\intercal & \boldsymbol{C} \end{pmatrix} \tag{14}$$

Let us calculate the first derivatives of $\pi(\boldsymbol{D}, \mathbf{v})$:

$$\frac{\partial \pi}{\partial v_a} = D^{-1}_{a\nu} v_\nu + D^{-1}_{\nu a} v_\nu \tag{15}$$

$$\frac{\partial \pi}{\partial D_{ij}} = -v_\mu D^{-1}_{\mu i} D^{-1}_{j\nu} v_\nu \tag{16}$$

and the elements of the Hessian:

$$A_{ab} = \frac{\partial^2 \pi}{\partial \mathbf{v}_a \partial \mathbf{v}_b} = 2D^{-1}_{ab} \tag{17}$$

$$B_{a,ij} = \frac{\partial^2 \pi}{\partial v_a \partial D_{ij}} = -[D^{-1}_{ai} D^{-1}_{j\nu} + D^{-1}_{\nu i} D^{-1}_{ja}] v_\nu \tag{18}$$

$$C_{ij,kl} = \frac{\partial^2 \pi}{\partial D_{ij} \partial D_{kl}} = v_\mu v_\nu [D^{-1}_{\mu k} D^{-1}_{li} D^{-1}_{j\nu} + D^{-1}_{\mu i} D^{-1}_{jk} D^{-1}_{l\nu}] \tag{19}$$

We can now proceed by verifying the condition expressed in Eq. (13).

$$(\mathbf{w}, \boldsymbol{P})\boldsymbol{\mathcal{H}}^{\pi}[\mathbf{v}, \boldsymbol{D}](\mathbf{w}, \boldsymbol{P}) = 2w_a D^{-1}_{ab} w_b - 2P_{ij} w_a [D^{-1}_{ai} D^{-1}_{j\nu} + D^{-1}_{\nu i} D^{-1}_{ja}] v_\nu \\ + P_{ij} P_{kl} v_\mu v_\nu [D^{-1}_{\mu k} D^{-1}_{li} D^{-1}_{j\nu} + D^{-1}_{\mu i} D^{-1}_{jk} D^{-1}_{l\nu}] \tag{20}$$

$$= 2\mathbf{w}\boldsymbol{D}^{-1}\mathbf{w} - 2\mathbf{w}\boldsymbol{D}^{-1}\boldsymbol{P}\boldsymbol{D}^{-1}\mathbf{v} - 2\mathbf{w}\boldsymbol{D}^{-1}\boldsymbol{P}^{\intercal}\boldsymbol{D}^{-1}\mathbf{v} \\ + 2\mathbf{v}\boldsymbol{D}^{-1}\boldsymbol{P}\boldsymbol{D}^{-1}\boldsymbol{P}\boldsymbol{D}^{-1}\mathbf{v} \tag{21}$$

Using that $\boldsymbol{P} \in \mathcal{S}_n$, meaning that we only consider directions within the subspace of symmetric matrices, and renaming $\mathbf{x} := \boldsymbol{P}\boldsymbol{D}^{-1}\mathbf{v}$, we simplify the above expression to

$$2[\mathbf{w}\boldsymbol{D}^{-1}\mathbf{w} - 2\mathbf{w}\boldsymbol{D}^{-1}\mathbf{x} + \mathbf{x}\boldsymbol{D}^{-1}\mathbf{x}] = 2(\mathbf{w} - \mathbf{x})\boldsymbol{D}^{-1}(\mathbf{w} - \mathbf{x}) \geq 0 \tag{22}$$

which holds due to $\boldsymbol{D}$ being positive definite. □

Proof of Lemma 2

First, let us note that the inequality $\mathbf{v}_{\mathrm{o}}(\boldsymbol{D}_{\mathrm{oo}})^{-1}\mathbf{v}_{\mathrm{o}} \leq \mathbf{v}\boldsymbol{D}^{-1}\mathbf{v}\ \forall_{\mathbf{v}\in\mathbb{R}^n}$ is equivalent to

$$\begin{pmatrix} (\boldsymbol{D}^{-1})_{\mathrm{oo}} - (\boldsymbol{D}_{\mathrm{oo}})^{-1} & (\boldsymbol{D}^{-1})_{\mathrm{oh}} \\ (\boldsymbol{D}^{-1})_{\mathrm{ho}} & (\boldsymbol{D}^{-1})_{\mathrm{hh}} \end{pmatrix} \succeq 0 \tag{23}$$

To simplify the notation let us introduce $\boldsymbol{Q} := \boldsymbol{D}^{-1}$. Since $\boldsymbol{D}$ is positive definite, we also have $\boldsymbol{Q} \succ 0$ and in particular $\boldsymbol{Q}_{\mathrm{hh}} \succ 0$. Rewriting the above condition in terms of $\boldsymbol{Q}$ we get:

$$\begin{pmatrix} \boldsymbol{Q}_{\mathrm{oo}} - [(\boldsymbol{Q}^{-1})_{\mathrm{oo}}]^{-1} & \boldsymbol{Q}_{\mathrm{oh}} \\ \boldsymbol{Q}_{\mathrm{ho}} & \boldsymbol{Q}_{\mathrm{hh}} \end{pmatrix} \succeq 0 \tag{24}$$

Using Schur's decomposition, we can write $(\boldsymbol{Q}^{-1})_{\mathrm{oo}} = (\boldsymbol{Q}_{\mathrm{oo}} - \boldsymbol{Q}_{\mathrm{oh}}(\boldsymbol{Q}_{\mathrm{hh}})^{-1}\boldsymbol{Q}_{\mathrm{ho}})^{-1}$. Substituting this in the equation above we get:

$$\begin{pmatrix} \boldsymbol{Q}_{\mathrm{oh}}(\boldsymbol{Q}_{\mathrm{hh}})^{-1}\boldsymbol{Q}_{\mathrm{ho}} & \boldsymbol{Q}_{\mathrm{oh}} \\ \boldsymbol{Q}_{\mathrm{ho}} & \boldsymbol{Q}_{\mathrm{hh}} \end{pmatrix} \succeq 0 \tag{25}$$

To check the positive semi-definiteness of this matrix we can apply it to an arbitrary vector $\mathbf{v} = (\mathbf{v}_{\mathrm{o}}, \mathbf{v}_{\mathrm{h}})$. The matrix in Eq. (25) is positive semi-definite if and only if

$$\mathbf{v}_{\mathrm{o}}\boldsymbol{Q}_{\mathrm{oh}}(\boldsymbol{Q}_{\mathrm{hh}})^{-1}\boldsymbol{Q}_{\mathrm{ho}}\mathbf{v}_{\mathrm{o}} + 2\mathbf{v}_{\mathrm{o}}\boldsymbol{Q}_{\mathrm{oh}}\mathbf{v}_{\mathrm{h}} + \mathbf{v}_{\mathrm{h}}\boldsymbol{Q}_{\mathrm{hh}}\mathbf{v}_{\mathrm{h}} \geq 0 \tag{26}$$

To simplify this expression let us substitute $\mathbf{w}_{\mathrm{h}} = \boldsymbol{Q}_{\mathrm{ho}}\mathbf{v}_{\mathrm{o}}$. This gives us:

$$\mathbf{w}_{\mathrm{h}}(\boldsymbol{Q}_{\mathrm{hh}})^{-1}\mathbf{w}_{\mathrm{h}} + 2\mathbf{w}_{\mathrm{h}} \cdot \mathbf{v}_{\mathrm{h}} + \mathbf{v}_{\mathrm{h}}\boldsymbol{Q}_{\mathrm{hh}}\mathbf{v}_{\mathrm{h}} \geq 0 \tag{27}$$

Finally we substitute $\mathbf{w}_{\mathrm{h}} = \boldsymbol{Q}_{\mathrm{hh}}\mathbf{x}$, $\mathbf{v}_{\mathrm{h}} = \mathbf{y}$, to get:

$$\mathbf{x}\boldsymbol{Q}_{\mathrm{hh}}\mathbf{x} + 2\mathbf{x}\boldsymbol{Q}_{\mathrm{hh}}\mathbf{y} + \mathbf{y}\boldsymbol{Q}_{\mathrm{hh}}\mathbf{y} \geq 0 \iff \tag{28}$$

$$(\mathbf{x} + \mathbf{y})\boldsymbol{Q}_{\mathrm{hh}}(\mathbf{x} + \mathbf{y}) \geq 0 \tag{29}$$

which holds, due to $\boldsymbol{Q}_{\mathrm{hh}}$ being positive definite. □

SUPPLEMENTARY REFERENCES

[1] Ghanta, A., Neu, J. C. & Teitsworth, S. Fluctuation loops in noise-driven linear dynamical systems. *Physical Review E* **95**, 032128 (2017).

[2] Mura, F., Gradziuk, G. & Broedersz, C. P. Nonequilibrium Scaling Behavior in Driven Soft Biological Assemblies. *Physical Review Letters* **121**, 038002 (2018).

[3] Gonzalez, J. P., Neu, J. C. & Teitsworth, S. W. Experimental metrics for detection of detailed balance violation. *Physical Review E* **99**, 022143 (2019).

[4] Gradziuk, G., Mura, F. & Broedersz, C. P. Scaling behavior of nonequilibrium measures in internally driven elastic assemblies. *Physical Review E* **99**, 052406 (2019).

[5] Frishman, A. & Ronceray, P. Learning force fields from stochastic trajectories. *Phys. Rev. X* **10**, 021009 (2020).

[6] Berman, G. J., Choi, D. M., Bialek, W. & Shaevitz, J. W. Mapping the stereotyped behaviour of freely moving fruit flies. *Journal of The Royal Society Interface* **11**, 20140672 (2014).
