# Supplementary material for: Learning the non-equilibrium dynamics of Brownian movies
Source: Nat Commun. 2020 Oct 23;11:5378. doi: 10.1038/s41467-020-18796-9 (PMC7585442; doi:10.1038/s41467-020-18796-9)
Supplement: Supplementary file 3 — Description of Additional Supplementary Files [file 41467_2020_18796_MOESM3_ESM.pdf]

Supplementary Movie 1

Description: Brownian movie of the two-beads system from Fig. 2 of the main text. Simulation parameters are also identical. The left bead has temperature T1=1.0, the right one T2=0.5.

Supplementary Movie 2

Description: Movie of the exact image force for the two-bead system shown in Supplementary Movie 1. The color map is the same as in the corresponding Fig. 2i.

Supplementary Movie 3

Description: Movie of the inferred image force for the two-bead system shown in Supplementary Movie 1. The color map is the same as in the corresponding Fig. 2i.

Supplementary Movie 4

Description: Brownian movie of the 5x5 patch in the 20x20 network of spring bonds with heterogeneous temperatures (Tcold=0.05, Thot=0.25). Simulation parameters are those of Fig. 3 of the main text.

Supplementary Movie 5

Description: Movie of the smaller area within Supplementary Movie 4 used for force inference.

Supplementary Movie 6

Description: Movie of the exact image force for the network shown in Supplementary Movie 5. The color map is the same as in the corresponding Fig. 3g.

Supplementary Movie 7

Description: Movie of the inferred image force for the network shown in Supplementary Movie 5. The color map is the same as in the corresponding Fig. 3g.

Supplementary Movie 8

Description: Brownian movie of the 5x5 patch in the 20x20 network of spring bonds with lower heterogeneous temperatures (Tcold=0.01, Thot=0.05) compared to Fig. 3 of the main text. The other simulation parameters are identical to those of Fig. 3 of the main text.

Supplementary Movie 9

Description: Movie of the smaller area within Supplementary Movie 8 (Low Temperature) used for force inference.

Supplementary Movie 10

Description: Movie of the exact image force for the network shown in Supplementary Movie 9 (Low Temperature). The color map is the same as in the corresponding Fig. 3h.

Supplementary Movie 11

Description: Movie of the inferred image force for the network shown in Supplementary Movie 9 (Low Temperature). The color map is the same as in the corresponding Fig. 3h.
